# Sporadic Nonautoimmune Neonatal Hyperthyroidism Due to A623V Germline Mutation in the Thyrotropin Receptor Gene

**DOI:** 10.4274/jcrpe.v2i4.168

**Published:** 2010-11-07

**Authors:** Zehra Aycan, Sebahat Yılmaz Ağladıoğlu, Serdar Ceylaner, Semra Çetinkaya, Veysel Nijat Baş, Havva Nur Peltek Kendirici

**Affiliations:** 1 Dr. Sami Ulus Women Health, Children’s Education and Research Hospital, Clinics of Pediatric Endocrinology, Ankara, Turkey; 2 Intergen Genetics Center, Clinical Genetics, Ankara, Turkey; +90 312 305 60 00+90 506 240 01 03zehraaycan67@hotmail.comPediatric Endocrinologist, Dr. Sami Ulus Women Health, Children\'s Education and Research Hospital, Clinics of Pediatric Endocrinology, Ankara, Turkey

**Keywords:** Thyrotropin receptor, nonautoimmune hyperthyroidism, germline mutation

## Abstract

Neonatal hyperthyroidism is a rare disorder and occurs in two forms. An autoimmune form is associated with maternal Graves' disease, resulting from transplacental passage of maternal thyroid−stimulating antibodies and a nonautoimmune form is caused by gain of function mutations in the thyrotropin receptor (TSHR) gene. Thyrotoxicosis caused by germline mutations in the TSHR gene may lead to a variety of clinical consequences. To date, 55 activating mutations of the TSHR gene have been documented. Fourteen cases with sporadic activating TSHR germline mutations have been described. Here we report a male infant with nonautoimmune hyperthyroidism due to an activating germline TSHR mutation (A623V), whose clinical picture started in the newborn period with severe hyperthyroidism. His parents did not have the same mutation. This mutation had been previously detected as a somatic mutation in patients with toxic adenomas. This is the first report of a sporadic case of nonautoimmune congenital hyperthyroidism associated with A623V mutation.

**Conflict of interest:**None declared.

## INTRODUCTION

Congenital hyperthyroidism is a rare disease and most cases are caused by transplacental passage of maternal thyrotropin receptor (TSHR) antibodies, which leads to transient hyperthyroidism in infants of mothers with Graves' disease (1). A persistent, nonautoimmune form of hyperthyroidism results from gain−of−function mutation in the TSHR gene. Heterozygous germline mutations in the affected subjects result in constitutive activation of the cyclic AMP (cAMP) pathway, which in turn stimulates the thyroid hormone production and thyrocyte proliferation (2).

Gain of function mutations are, by definition, dominant, and alteration of one allele is thus sufficient for generating the pathologic phenotype. Activating TSHR mutations can occur somatically in solitary toxic adenomas or toxic adenomas within multinodular goiters. Germline TSHR mutations give rise to autosomal dominant nonautoimmune hyperthyroidism or, in case of de novo mutations, to sporadic nonautoimmune congenital hyperthyroidism. The clinical features of sporadic nonautoimmune hyperthyroidism include earlier onset of thyrotoxicosis and more severe clinical symptoms, which are difficult to control as compared to familial cases (3). However, a severe course in familial nonautoimmune hyperthyroidism has also been reported (4, 5).

The clinical symptoms of nonautoimmune hyperthyroidism include variable severity of hyperthyroidism and goiter, absence of thyroid autoantibodies and absence of lymphocytic infiltration in the thyroid histology. The hyperthyroid state typically relapses following the cessation of antithyroid drugs. In some cases antithyroid drug treatment fails to control the hyperthyroidism at long−term follow−up and thyroidectomy or radioiodine therapy become necessary.

De novo activating TSHR germline mutations have been previously reported in 14 cases as the cause of sporadic congenital nonautoimmune hyperthyroidism and these cases have ten different TSHR germline mutations (6, 7). Almost all mutations are located in the transmembrane domain of the TSHR protein, which is encoded by exon 10. So far, only one mutation has been identified in the extracellular domain (8).

In this report, we present a Turkish boy with sporadic congenital hyperthyroidism who presented with severe symptoms of hyperthyroidism in early infancy and a heterozygous TSHR germline mutation. Until now, this mutation has not been reported in sporadic cases of nonautoimmune congenital hyperthyroidism.

## CASE REPORT

Our patient was a male infant delivered at the 39th week of an unremarkable gestation as the first child of unrelated Turkish parents. Birth weight was 3500 g. The parents reported that during the neonatal period, the infant had suffered from poor weight gain, diarrhea, excessive sweating, and irritability. At the age of 6 months he was referred to our hospital. At this time, his weight was 5400 g (3−10th percentile), total weight gain from birth was 1900 g, length was 65 cm (25−50th percentile) and head circumferences 42 cm (10 −25th percentile). Blood pressure was measured as 95/55 mmHg with a heart rate of 138beats/min. He had goiter and exophthalmos. Bone age was significantly advanced and corresponded to three years of age. Laboratory tests confirmed hyperthyroidism with a TSH level of 0.05 μIU/mL (0.4−4), free T4 >6 ng /dL (0.8−1.9), free T3 of 12.9 pg /mL (1.6−4.7), total T4 >24 μg/dL (4.5−12.5), total T3 of 4.1 ng /mL (0.7−1.9). TSHR, thyroid peroxidase (TPO) and human thyroglobulin (TG) antibodies were negative in the patient and his mother. Thyroid ultrasound showed diffuse enlargement of the thyroid gland.

The patient was started on propylthiouracil and propranolol. After four weeks of propylthiouracil treatment a euthyroid state was reached. Thyroid hormone levels continued to be normal during a six−month period of follow−up with antithyroid therapy. When the treatment was stopped by the family at the age of 12 months, the hyperthyroid state relapsed with markedly elevated total and free thyroid hormone and suppressed TSH levels. Propylthiouracil treatment was reinitiated.

**Methodology and Results of the Genetic Analysis**

EDTA−blood was drawn from the index patient and his parents for extraction of genomic DNA from peripheral blood leucocytes using spin column method. All the exons of the gene were sequenced by the primers placed to the neighboring introns. Genetic analysis of genomic DNA extracted from the patient’s peripheral blood sample showed a heterozygous point mutation at codon 623 in exon 10 that codes the transmembrane domain of the TSHR. This mutation results in an amino acid substitution of alanine to valine. The mutation was not detected in the peripheral blood samples of the parents.

Thus, the molecular analysis of the patient's TSHR gene revealed a de novo heterozygous mutation (A623V) at codon 623 in exon 10, at codon 623, which leads to an amino acid substitution from alanine to valine at that position. The mutation was not detected in his mother or father.

During 18 months of follow−up, the patient remained euthyroid on propylthiouracil treatment. Table 1 shows clinical and laboratory findings of the patient at admission and at follow−up.

**Table 1:**
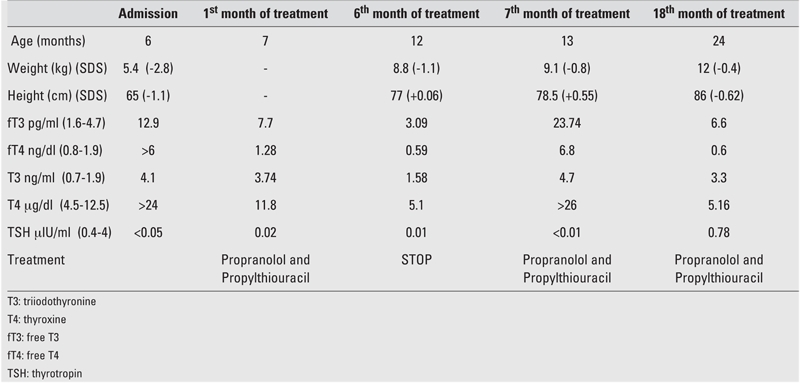
The clinical and laboratory findings of the patient at admission and at follow−up

## DISCUSSION

Here we report a case of sporadic congenital nonautoimmune hyperthyroidism with an activating TSHR germline mutation in a Turkish boy. The mutation occurred de novo, since neither parent harbored the TSHR mutation. In addition, both parents had normal test results of thyroid function. The possibility of gonadal mosaicism was discussed with the parents to inform them about the low recurrence risk in further pregnancies.

This mutation has been described previously as a somatic mutation in toxic nodules in familial nonautoimmune hyperthyroidism (4, 9), but the germline heterozygous mutation (A623V) at codon 623 in exon 10 found in our patient, has not been reported in sporadic cases.

The first patient with sporadic nonautoimmune congenital hyperthyroidism due to a germline mutation was described by Kopp et al in 1995 (10). To date, fourteen cases with sporadic activating TSHR germline mutations have been described associated with 10 different mutations. Table 2 summarizes the data of 14 previously reported cases of nonautoimmune hyperthyroidism resulting from sporadic TSHR germline mutations, highlighting the variability of the clinical consequences of this condition.

Although there are reports of patients with relatively severe courses also in cases of familial nonautoimmune hyperthyroidism (4, 5), inherited TSHR mutations usually present later in life with rather mild clinical manifestation. Sporadic TSHR mutations were found to cause severe thyrotoxicosis and goiter with onset in the neonatal period or infancy. Almost all reported cases became symptomatic within the first year of life, like our patient (11). In sporadic congenital nonautoimmune hyperthyroidism, patients with the same TSHR germline mutations display variable phenotypes, particularly with respect to the onset of thyrotoxicosis.

Our patient has clinical features similar to other cases of sporadic nonautoimmune congenital hyperthyroidism described in the literature. Severe neonatal hyperthyroidism and advanced bone age characterized the phenotype of our patient. Recurrence of thyrotoxicosis occurred when antithyroid drug therapy was stopped.

Our patient was born at term, while more than half of the previous patients were born preterm. The most prominent features of the previously reported cases were preterm birth and associated symptoms like developmental delay, irritability, diarrhea and excessive sweating. Our patient, although born at term with a normal birth weight, had poor weight gain, diarrhea, excessive sweating, and irritability.

The size of the thyroid gland is also variable in patients with sporadic nonautoimmune hyperthyroidism. In some patients, the initial manifestation of the constitutively active TSHR mutation is hyperthyroidism without any increase in the gland mass (4, 6, 8, 11, 12, 13, 14, 15), whereas in others (7, 10, 16, 17, 18, 19), goiter development may precede the onset of the clinical findings of hyperthyroidism. Our patient had grade II goiter at the time of diagnosis at age six months. Only two cases have been reported with a nodular goiter. These patients were 7 and 8.7 years old (10, 12). Craniosynostosis is another prominent feature, which has been observed in seven of fourteen previously reported cases. This feature was not present in our patient.

Symptoms of developmental impairment such as mental retardation, hydrocephalus and speech disturbance have been reported in 7 cases of sporadic nonautoimmune hyperthyroidism (4, 10, 11, 12, 13, 17, 18). Our patient’s development was compatible with his age. This finding could be due to a relatively early diagnosis and aggressive therapy with antithyroid drugs.

Accelerated bone age, a common finding in these cases, was also evident in our patient.

In addition, various clinical findings such as jaundice, hepatosplenomegaly, thrombocytopenia (19) and severe respiratory symptoms such as apnea, asphyxia (6, 12, 15, 19) have been reported in association with this condition.

Many of the cases were reported soon after their diagnosis. Longer follow−up findings have been reported in 8 patients aged between 6 and 22 years (7, 8, 10, 11, 12, 13, 15, 18).

Different therapeutic protocols had been used in the patients reported in the literature, even in those with similar mutations. Total thyroidectomy had been performed in three patients described by Gruters et al (8), Schwab et al (4), and Esapa et al (16). The patients described by Holzapfel et al (13) and Bircan et al (7) underwent a neartotal thyroidectomy after a long−term antithyroid treatment. The subjects described by Lavard et al (12) and by Kopp et al (10) had received I131 therapy in addition to subtotal thyroidectomy. The patient described by Nishihara et al. (11) had received both medical and I131 therapy. In other cases (6, 15, 17, 19), only medical therapy had been given. Propylthiouracil is a drug, which carries the risk of causing severe hepatic insufficiency and it is not a first choice therapy option for children. Methimazole is currently recommended as the first line of antithyroid drug treatment. Total thyroidectomy is planned in our patient.

An association between genotype and phenotype in presence of constitutively active TSHR mutations is difficult to establish since one and the same mutation may lead to variability in the clinical course and onset of hyperthyroidism (13, 14).

Although congenital nonautoimmune hyperthyroidism is rare, in patients with neonatal hyperthyroidism with negative antibodies, TSHR mutations should be included in the differential diagnosis. Diagnosing nonautoimmune hyperthyroidism due to activating TSHR mutation is of great importance for both patient management and genetic counseling.

The early identification of individuals with nonautoimmune hyperthyroidism is important, because delayed or inadequate treatment of hyperthyroidism might lead to irreversible consequences such as mental retardation. Thus, aggressive therapy that may include surgery and ablation by radiotherapy should be applied promptly.

**Table 2:**
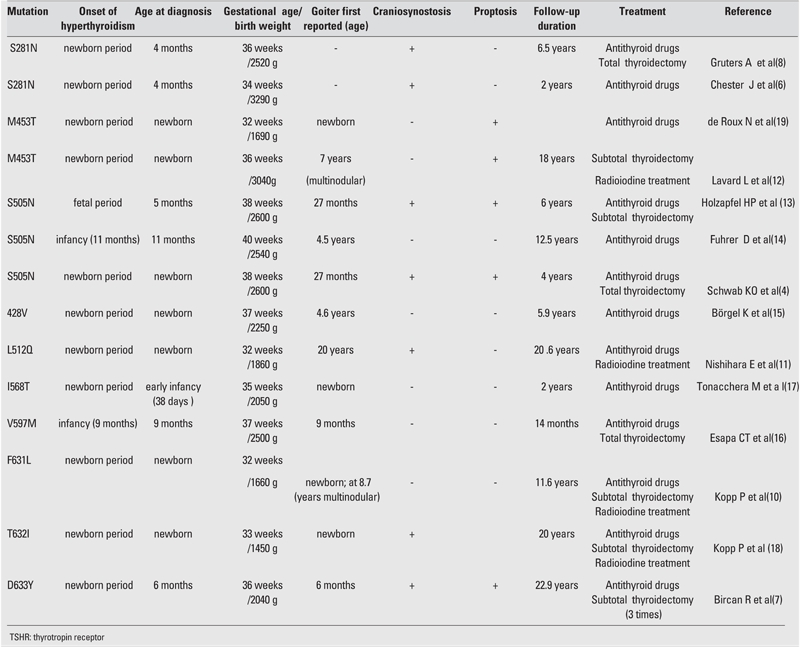
Summary of clinical and genetic information of 14 previously reported cases of non−autoimmune hyperthyroidism resulting from sporadic TSHR germline mutations

## ACKNOWLEDGEMENT

We are indebted to Gilbert Vassart at the Universitè Libre De Bruxelles for the genetic analysis of mutations in the TSHR gene.
